# Polymer-Modified Fertilizers for Mitigating Strawberry Root Burn

**DOI:** 10.3390/polym16202950

**Published:** 2024-10-21

**Authors:** Ibragim Bamatov, Kirill Perevertin, Nadezda Vasilyeva

**Affiliations:** V.V. Dokuchaev Soil Science Institute, Moscow 119017, Russia; ibragim-1991@mail.ru (I.B.); perevertink@mail.ru (K.P.)

**Keywords:** soil, nutrients, controlled release, electrical conductivity, sustainable agriculture, environmental impact

## Abstract

Polymer-modified fertilizers (PMFs) with prolonged nutrient release present a promising solution to address the challenges associated with conventional fertilization practices, particularly for sensitive crops such as strawberries. This study investigates the effectiveness of biodegradable PMFs in maintaining nutrient availability at optimal levels while minimizing root burn and nutrient losses. In a factorial field experiment, we obtaineda total of 3780 sets of parallel measured time series for soil EC, moisture, and temperature as well as two sets of harvest data to evaluate the impact of varying concentrations of polyvinyl alcohol (PVA) on the nutrient release rates from complex NPK fertilizer and monoammonium phosphate. Results indicate that polymer modifications significantly slow down nutrient release, leading to optimal salt levels and maximizing yield while remaining low enough to prevent the risk of root burn (EC of soil solution below 1 mS/cm). Consequently, the application of PMFs enhances strawberry yield surplus (on average 2.8 times in the second harvest) by ensuring a steady supply of nutrients throughout the growing season without inducing stress, which reduces the yield by nearly half. This research provides valuable insights into the development of more effective fertilization strategies for strawberry cultivation and other sensitive crops using PMFs.

## 1. Introduction

The global strawberry harvest exceeds nine million tons annually [1]. Major producers of strawberries include China, the USA, Mexico, Egypt, Turkey, Spain, Brazil, and Russia [2]. To maintain such production levels, substantial amounts of mineral fertilizers are required. In 2022, global agricultural consumption of mineral fertilizers reached approximately 195 million tons of nitrogen, 65 million tons of phosphorus, and 75 million tons of potassium [3]. However, the efficiency of applied fertilizers diminishes due to their rapid dissolution, which occurs faster than plants can utilize them. Estimates suggest that the uptake rate for standard forms of mineral fertilizers by plants is only 30–35% [4]. Most transformations of nutrients in the soil or soil solution are influenced by their concentration. Consequently, any excess nutrients that are not taken up by plants may undergo three types of processes that diminish their availability: microbial processes, such as nitrification, denitrification, and immobilization; chemical processes, including exchange, fixation, precipitation, and hydrolysis; and physical processes, such as leaching, runoff, and volatilization [5,6].

These non-productive losses result in low nutrient use efficiency, leading to increased interest in the development of prolonged-release fertilizers [7,8,9,10,11,12,13]. Additionally, these losses pose environmental hazards by releasing greenhouse gases, such as N_2_O from denitrification, or by contaminating groundwater and nearby water bodies with salts [14,15,16,17,18]. The intensive use of mineral fertilizers can also contribute to soil degradation, characterized by acidification and reductions in organic matter, microbial biomass, and biodiversity [19,20]. Additionally, the periodic application of rapidly soluble fertilizers causes fluctuations in nutrient concentrations, oscillating between deficiency and toxicity levels [4].

A less obvious yet well-recognized and significant negative consequence of the rapid dissolution of dry mineral fertilizers is the potential for root burn in plants [21,22,23]. This stress occurs when salt concentrations in the soil solution exceed critical levels. The threshold electrical conductivity (EC) values at which root burn can happen vary depending on the soil type and the specific crop. For most crops, root burn may begin at EC levels above 2–3 dS/m in the saturated soil extract [24]. This corresponds to salt concentrations of approximately 1300–2000 mg/L. High salt content generates elevated osmotic pressure in the soil solution, making water energetically bound and difficult for roots to absorb. As a result, even when soil moisture is adequate, plants may suffer from drought stress. Additionally, water may start to move from the roots outward in an effort to dilute the soil solution. For particularly sensitive crops such as garden strawberries, root burn can occur at EC levels above 1 dS/m of saturated soil extract [25,26]. EC reflects the dynamics of salt concentrations in soil solutions and is a practical, easily measurable property, particularly important for salt-sensitive crops. However, there is a notable gap in the literature regarding the threshold soil EC values above which the risk of root burn occurs in plants. Furthermore, EC is typically reported as values for saturated soil paste, which is useful for assessing total salt content but does not accurately represent the concentration of salts in the soil solution that roots actually contact, as this value is highly dependent on soil moisture levels. Therefore, even temporary high concentrations of salts in the soil beneath sensitive crops can lead to stunted plant growth due to root burn, resulting in reduced yields. To adequately assess the risk of root stress from salt concentrations in the soil solution, parallel measurements of soil EC and actual volumetric water content are necessary to derive the EC of the soil solution.

Despite the long-standing awareness of this issue, scientific literature examining soil electrical conductivity and fertilizer efficiency primarily focuses on non-productive fertilizer losses resulting from rapid dissolution. There has been limited research on determining the electrical conductivity levels for different crops and their growth stages at which root burn significantly impacts yields. Additionally, studies on the recovery time for plants from stress and the subsequent restoration of productivity, particularly in remontant garden strawberries, are scarce. In practical agricultural settings, soil salt concentrations can be easily monitored through EC measurements [27]. Leveraging this data can greatly improve our understanding of the mineral fertilizer concentrations required to achieve the potential of various cultivars while ensuring root safety across different crops.

One approach to reduce the dissolution rate of mineral fertilizers is to modify them into controlled-release fertilizers (CRF) or slow-release fertilizers (SRF). The European Committee for Standardization (CEN) has established the following criteria for CRFs: (i) less than 15% of the nutrients (e.g., urea) should be released within 24 h; (ii) less than 75% of the nutrients should be released within 28 days; and (iii) more than 75% of the nutrients should be released within the specified release time [28]. CRFs are created by applying coatings, such as polymer or hydrogel films, to base fertilizers or by embedding the fertilizer in a polymer matrix with cross-linking. In this case, the release is slowed as it occurs through diffusion via the swollen membrane, micro-cracks in the membrane, or the polymer matrix [4,29]. The release rate is predictable, primarily depending on environmental temperature and humidity; thus, thicker coatings result in slower release rates. Higher temperatures accelerate diffusion through the polymer layer or matrix, while also promoting plant growth and nutrient uptake. For strawberries, air and soil temperatures have been identified as key predictors of growth [30].

SRFs, on the other hand, consist of natural or synthetic biodegradable nitrogen compounds. For instance, synthetic SRFs are produced through complex reactions of large molecules with fertilizer substances, and in this case, the further release of nutrients occurs through the breakdown of the carrier molecule either via hydrolysis or enzymatically. Therefore, the release rate of nutrients from SRFs, unlike CRFs, is not well-predicted and depends heavily on various environmental factors such as temperature, humidity, acidity, and microbial activity [31,32,33,34]. When developing prolonged-release fertilizers, it is preferable for their polymer modifiers to be biodegradable, as this ensures safety for humans and prevents accumulation in soils. Natural biodegradable polymers commonly used to slow the release of mineral fertilizers include starch, alginate, cellulose, chitin, chitosan, lignin, hemicellulose, guar gum, xanthan gum, and carrageenan. Examples of synthetic biodegradable polymer coatings include polyvinyl alcohol (PVA), polyvinyl chloride, polyacrylamide, rubber, polyurethane, polyethylene oxide, polyethylene glycol, polyacrylate, and polyacrylamide [35,36]. The rate of abiotic or biotic degradation is controlled by the composition of the coating or polymer matrix, as demonstrated in the case of urea modification [37].

For example, the use of combinations of PVA with chitosan leads to denser binding due to the formation of hydrogen bonds between chitosan and PVA and also exhibits excellent water swelling capacity. This two-layer structure ensures the strength of the coating against swelling, thus preserving controlled phosphorus fertilizer [38]. The effectiveness of polymer membranes that combine PVA with sodium alginate for coating fertilizers and ensuring prolonged nutrient release has been established [39]. Additionally, the combination of PVA with nanocrystalline cellulose has been used to create water-soluble fertilizer coatings with slow-release and moisture-retaining properties [40]. A formulation of PVA combined with starch has also been developed, showing good swelling and moisture retention characteristics, along with a significant positive impact on spinach yield [41].

Biodegradable polymers have also demonstrated improved properties when used in combination with biostimulants [42]. Overall, prolonged-release fertilizers can enhance various yield quality indicators. For instance, positive effects have been observed for leaf length and chlorophyll content in tuberose onion [43], spring wheat [44], Gluten Deformation Index in winter wheat [8], root activity, grain number, and grain weight in spring corn [45], as well as the number of ears, grains per ear, grain weight per ear, and germination of winter wheat [46]. The duration of action of prolonged-release fertilizers can reach up to 16 months, as seen with Multicote, depending on the thickness of the polymer coating, and up to 24 months with SK Cote [47,48]. This extended release period is particularly relevant in reducing the number of necessary fertilizer applications throughout the year, especially in year-round crop cultivation or no-till technologies, where a large amount of fertilizer must be applied at once at the beginning. This reduces soil load and labor costs. For example, remontant strawberry varieties can yield three harvests with a single application of prolonged-release fertilizers. Well-known examples of commercially available fertilizers of this type include Osmocote Pro, Tagrow, Planton Cote, Gro-Sure, Floranid, Polyon, and others.

CRFs currently dominate the market, and are projected to grow by about 50% in the next five years [49]. In summary, CRFs based on biodegradable polymers offer several advantages:

- They enhance fertilizer efficiency by reducing losses through controlled release rates that align with plant consumption. The polymer coating can protect nutrients from biological degradation and losses due to volatilization. This improves nutrient use efficiency while reducing environmental pollution from excess runoff or leaching of fertilizers. Possible savings on mineral fertilizers can reach 20–30%.
- They mitigate the risk of root burn by preventing spikes in fertilizer concentration, thereby promoting healthy plant growth and the realization of varietal potential. In addition to increasing yield, quality may also improve as plants experience no stress during development, receiving optimal nutrient amounts throughout the growing season.
- They enhance soil moisture retention, as some biodegradable polymers, such as polyacrylamide (PAM) and starch-based hydrogels, can absorb and retain large volumes of water. When used to coat fertilizers, these polymers can improve the soil water holding capacity, especially in arid conditions. This helps plants better withstand drought periods and increases water use efficiency.
- They improve soil quality through the decomposition of polymer coatings in the soil, which replenishes the pool of organic matter that serves as a buffering and structuring factor for soil fertility, supporting healthy plant growth.
- The longer shelf life of CRF reduces storage costs for mineral fertilizers.

One of the primary challenges in developing and applying prolonged-release fertilizers is identifying compositions and modification forms that ensure stable nutrient release at levels safe for plants over specified time intervals. The aim of this study was to evaluate the effectiveness of our developed polymer modification of azofoska and monoammonium phosphate (MAP) fertilizers with varying concentrations of PVA on the yield of garden strawberries and soil properties.

The initial hypothesis proposed that the plant use of CRFs would be effective due to their slow nutrient release and reduced non-productive losses. This was expected to help maintain higher nutrient concentrations during the formation of the second harvest of remontant garden strawberries with a single application.

To assess the impact of polymer modification on the nutrient release rate from fertilizers and, consequently, on yield, a factorial field experiment was conducted to grow garden strawberries using various nutrient systems. The nutrient systems included a comparison of the control, commercial CRF, and our own polymer modifications of the fertilizer classified as CRF with varying concentrations of the polymer. The study analyzed yield alongside time series data for soil electrical conductivity (EC_b_), soil moisture, and soil temperature over two growing seasons.

## 2. Materials and Methods

### 2.1. Preparation of Polymer-Modified Fertilizer (PMF) with 5 and 10% of Polymer

**Figure 1:**
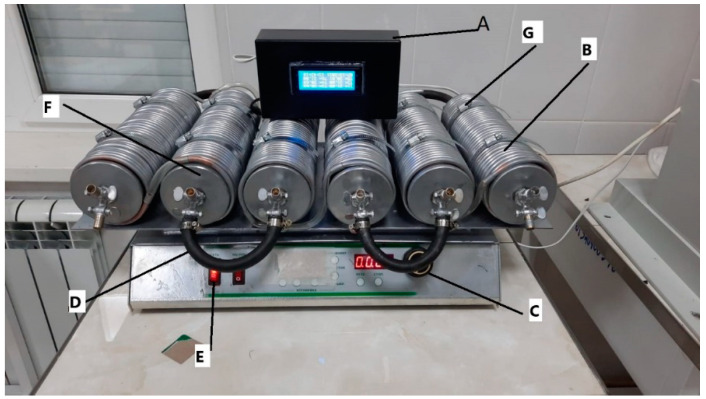
General view of the reactor. The reactor comprises several key components: the Information Processing Receiver (**A**) features a temperature control system for each stage; the Copper Heating/Cooling Line (**B**) includes outputs for connection to Hubert furnaces; the Horizontal Movement Speed Adjustment (**C**) is equipped with a display that indicates the number of movements per minute based on a motion sensor; the Reactor On/Off Button (**D**) is located next to the switch for the autonomous heating and cooling system; the Carbon Fiber Hose (**E**) connects the reactor stages, facilitating six mixing stages; the Reactor Pipes (Stages) (**F**) have advanced lid designs for optimized sealing; and finally, the Holders for Reactor Pipes (Stages) (**G**) provide structural support.

The PMFs were obtained at the research laboratories of the Federal State Budgetary Educational Institution of Higher Education Chechen State University using our previously patented V-star reactor, which allows producing PMF forms based on traditional mineral fertilizers and biodegradable polymers. Complex NPK fertilizer (Azofoska) and MAP (peoduced by LLS “Uralchim”, Moscow, Russia) were used as base mineral fertilizers. Polyvinyl alcohol (PVA) was used as the biodegradable polymer and citric acid was used as the cross-linking agent [50]. The V-star flow chemical reactor, shown in Figure 1, is a 6-stage coil, where each stage can continuously mix liquids, and each is attached to the next stage via a flexible connector (tube) made of hydrocarbon fiber to enable reactions at high-temperature regimes.

The NPK fertilizer was dissolved in water at a 1:1 ratio, heated to 50 °C while stirring, while simultaneously at 70 °C, a biodegradable polymer PVA was stirred with water using a stirrer at a speed of 600 rpm until a gel-like suspension formed. Then the mixtures were combined, 10% citric acid was added by weight of the biodegradable polymer, the speed was increased to 800 rpm, the mixture was heated to 80 °C, cooled for 24 h, aged for 72 h at 5 °C, filtered, dried for 72 h, and tableted. PVA constituted 5 and 10% by weight of the fertilizers. We used PVA 17-99 (20 mesh) manufactured by LLC “Titan”, Moscow, Russia.

**Figure 2:**
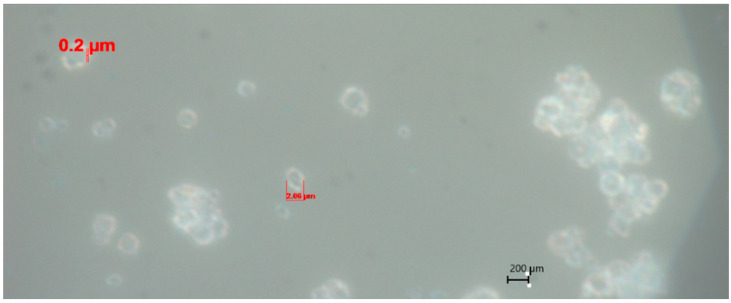
Microscope image of 10% PVA polymer-coated Azofoska (at 100× magnification, dark field mode) [51].

Preliminary studies showed that the size of the polymer coating layer on Azofoska (N16:P16:K16) fertilizer prepared with 10% of PVA polymer was 20 μm [51]. The thickness of the layer was measured under a microscope at 100× magnification (Figure 2). Fertilizers with the resulting polymer coating had the appearance of reddish, dry tablets (for ease of use).

PMF based on MAP was tested (April–July 2021) on various varieties of apple (Geneva Early) and plum (Kabardinskaya Rannyaya). Experimental work was carried out in the Chechen State University and LLC Scientific and Production Company “Gardens of Chechnya”. The experiment showed that MAP with a biopolymer coating surpassed the Tagra (TAGROW) analogue in terms of indicators such as the number of stems, changes in soil agrochemical parameters, fruit weight, and yield [51].

PMF based on Azofoska was tested on berry plants (2-year-old strawberry plants, Elizaveta variety) under greenhouse conditions and surpassed the CRF analogue TAGROW in the following indicators: number of opened inflorescences, volume of the plant root system, plant stress resistance, and berry yield [52].

Preliminary experiments with different polymer contents showed that the PMFs with a polymer content of 5% and 10% are of interest for further study.

### 2.2. Field Experimental Design

The experiment on the effects of PMF-based nutrition systems on the yield of garden strawberries was conducted in the greenhouse complex of ‘Gardens of Chechnya’ in Kurchaloy. The strawberry plants were obtained in vitro [53]. Virus-free two-year-old material was planted in trays with a drip irrigation system, with a spacing of 15 cm between plants in a greenhouse (dim. 20 m × 5 m) on 30 March 2023. The experiment utilized a randomized complete block design with three independent replications and involved five nutrition systems: control (no fertilizer), a traditional system (non-polymer modified components), a commercial CRF (OSMOCOTE, hereinafter referred to as “analogue”), and two developed nutrition systems with different levels of polymer modification, described above. The traditional nutrition system included two tablets of the unmodified Azofoska and one tablet of unmodified MAP for every four plants. The developed nutrition systems contained the same fertilizers in a polymer-modified form to reduce the release rate. The PVA content was 5% in the first and 10% by weight in the second one, calculated for 3 and 6 months of prolonged action, respectively, hereafter referred to as “PMF-5” and “PMF-10”.

**Figure 3:**
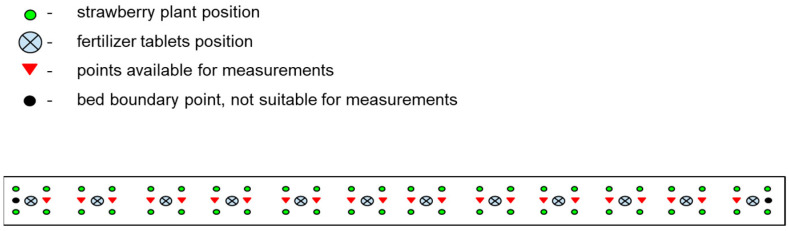
Scheme of the experimental bed shows the arrangement of plants, fertilizers, and points available for random replicated measurements of soil properties (temperature, moisture, EC).

The total amount of active substance in three tablets of fertilizer in each nutrition system was consistently 8.4 g for four plants, or 2.1 g of active substance per plant. Each tablet of Azofoska 16:16:16 contained 0.8 g of each of the NPK element. Each MAP tablet contained 0.6 g of nitrogen and 3 g of phosphorus. The fertilizer dosage per plant was calculated based on practical experience in the region and recommendations from the Ministry of Agriculture of Russia, which suggest 80–85 kg of active substance per hectare when planting 35–40 thousand plants per hectare in field conditions, averaging 2.1 g of active substance per plant. The tablet fertilizers were buried in the soil on 6 April 2023, according to the typical bed scheme shown in Figure 3. The fertilizer tablets were placed in a stack at the center of the square, with four strawberry plants positioned at the corners, for which they were intended.

The experimental options included combinations of each nutrient system with three remontant strawberry varieties (Profusion, Irma, and Elizaveta). The experiment aimed to achieve two harvests. Different varieties of garden strawberries were selected based on practical considerations to mitigate the risks of yield loss, identify the most productive variety, and exclude the possibility of obtaining a specific response from a particular variety to the prolonged nutrient supply in the results. The five nutrition systems and three strawberry varieties resulted in fifteen experimental variants. Each variant had 3 independent field replications, leading to a total of 45 experimental plots. When analyzing data unrelated to the yield of a specific strawberry variety, we utilized the experimental variants of the three varieties as additional independent replications of the experiment.

The experimental beds were filled with soil collected from a nearby location. The soil was characterized as southern chernozem, carbonate, with a clayey texture, a density of 1.2 g/cm^3^, a water extract pH of 8.3 ± 0.1, a salt extract pH of 7.5 ± 0.1, and an organic matter content of 4.0 ± 0.7%. At the end of the growing season, the soil contained 202 ± 30 mg/kg of mobile phosphorus forms and 54 ± 10 mg/kg of mobile potassium forms, indicating very high phosphorus availability and medium potassium availability for this type of soil.

### 2.3. Measurements

#### 2.3.1. Strawberry Yield

**Figure 4:**
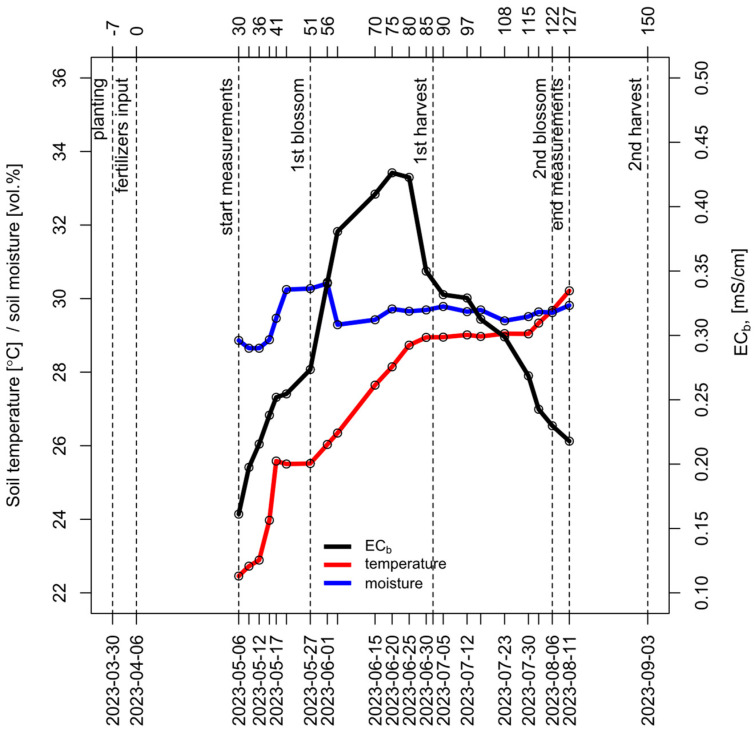
Key events of the experiment, including planting, fertilizer input, flowerings, harvests, and dates of measurements. The graph reflects the general picture of changes in temperature and soil moisture (left axis) and soil EC (right axis) throughout the experiment (averaged across all experimental variants).

The duration of the experiment was approximately 4 months (120 days), during which 2 harvests of garden strawberries were collected. Harvesting was conducted twice: on 2 June 2023 and 3 September 2023. The average yield per plant was measured as an average value for each bed (total yield/number of plants). Thus, for each experimental variant, the yield was determined in three field replications. The overall timeline of all experimental dates is presented in Figure 4.

#### 2.3.2. Collection of Time Series for Soil Moisture, Temperature and EC

Soil measurements started on 6 May 2023 with the final measurements made on 11 August 2023. Measurement days are counted from fertilizer input as day 0. The timing for regular measurements was set to occur approximately half an hour after the completion of the daily irrigation, which took place at 4 PM. This schedule was established to ensure optimal moisture conditions for measuring electrical conductivity (EC), as it is recommended that EC be assessed at a volumetric moisture content of at least 25% [54].

All measurements were performed using a compact field digital meter, the YY-1000 3-in-1, VitaFerma, Beijing, China, which was calibrated with standard solutions having EC values of 1.413 mS/cm and 12.880 mS/cm. In each of the 45 experimental beds, replicate measurements were taken at four sampling points to account for soil heterogeneity. On each measurement day, the sampling points were randomly selected from those marked with a red triangle in the scheme (Figure 4) to minimize the influence of any specific plant’s condition on the overall series of measurements.

The frequency of measurements was 2–3 times per week, resulting in a total of 21 measurement dates, as shown in Figure 4. For each of the fifteen experimental variants (combination of nutrient system and variety), four simultaneous measurements were taken at each of the three independent field replications of the experiment at every measurement time point. These measurements included soil EC, temperature, and moisture.

During the growing season, a total of 3780 sets of parallel measurements were collected, comprising time series data for soil EC, moisture, and temperature (5 nutrient systems × 3 varieties × 3 independent field bed replications × 4 replicate bed measurements × 21 time points), along with two sets of 90 yield values in total (5 nutrient systems × 3 varieties × 3 independent field bed replications × 2 harvests).

#### 2.3.3. Infrared Spectrometry of PMF

Infrared transmission spectra were recorded using a Vertex 70 FT-IR spectrometer (BRUKER, Bremen, Germany) over the wavenumber range of 4000–400 cm^−1^. Samples for transmission measurements were prepared by finely grinding Azofoska and mixing it with KBr powder in a 1:300 ratio, followed by pressing into tablets. The transmittance spectra spanned a frequency range of 369.3559 to 3998.302 wavenumbers, with a resolution of 0.96437 wavenumbers per data point. FT-IR absorbance spectra were then calculated as −log_10_ (Transmittance) and baseline corrected using the asymmetric least squares method.

#### 2.3.4. Nutrient Release Rate Analysis of PMF

To investigate nutrient release patterns from PMF into the soil environment, samples of polymer-modified Azofoska and monoammonium phosphate with PVA concentrations of 5 and 10% were analyzed. The analysis was conducted under controlled laboratory conditions at 25 °C and in a greenhouse environment at approximately 35 °C, allowing for natural temperature fluctuations, with four replications for each condition. The PMFs were placed in pots containing a model soil substrate (“3D”) at a relative humidity of 55% for a duration of 26 weeks. Weekly monitoring of the tablet forms of the fertilizers was performed, and the rate of mineral substance release was determined based on the weight change of the tablets over one week.

#### 2.3.5. Data Processing and Statistical Analysis

All data processing, including the analysis of infrared (IR) spectra, clustering, principal component analysis (PCA), statistical tests, and graph construction, was performed using the open-source R software package v.4.1.3 [55]. Mean values presented in the text are accompanied by the standard error of the mean.

## 3. Results

### 3.1. Strawberry Yield

The overall productivity of garden strawberries was assessed during the experiment. The average yield of the second harvest was significantly lower, at 53 ± 4 g/plant, compared to the first harvest, which yielded 82 ± 5 g/plant across all studied varieties and nutrition systems. This decline in yield was characteristic of the growing season under the specific cultivation conditions and was consistent regardless of the variety or nutrient supply system employed.

#### 3.1.1. Comparison of Varieties by Productivity

**Figure 5:**
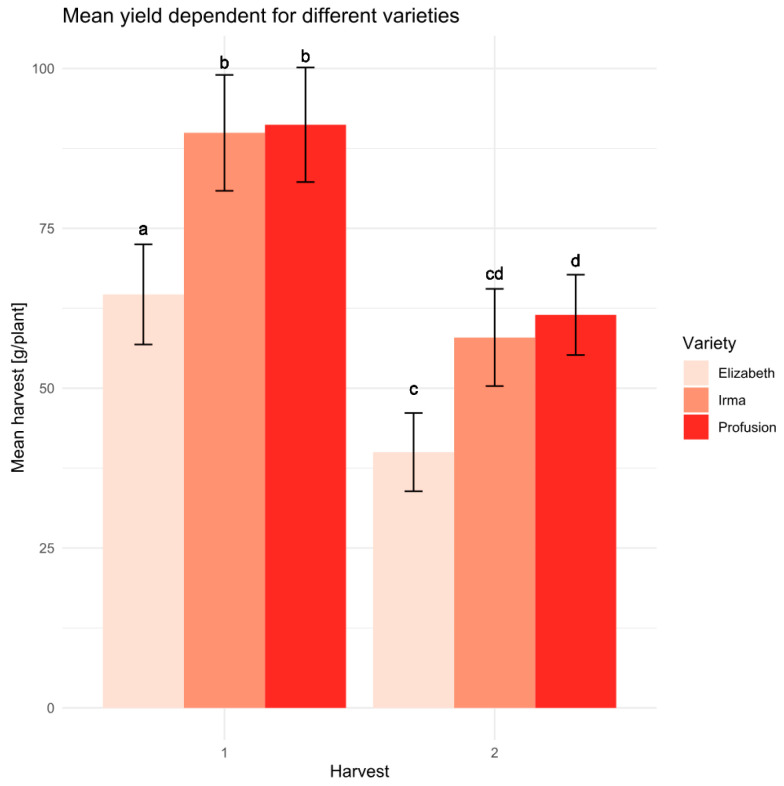
Average yield values with a standard deviation for the studied varieties at the two harvests. Letters indicate statistical differences according to the Student’s *t*-test at a confidence level > 0.95 (sample size *n* = 15).

Among the varieties tested, the Elizaveta variety exhibited a statistically significant lowest productivity compared to the other two varieties in the experiment. The Irma and Profusion varieties showed no significant differences in productivity (Figure 5).

#### 3.1.2. Influence of Nutrition Systems on Strawberry Yields

**Figure 6:**
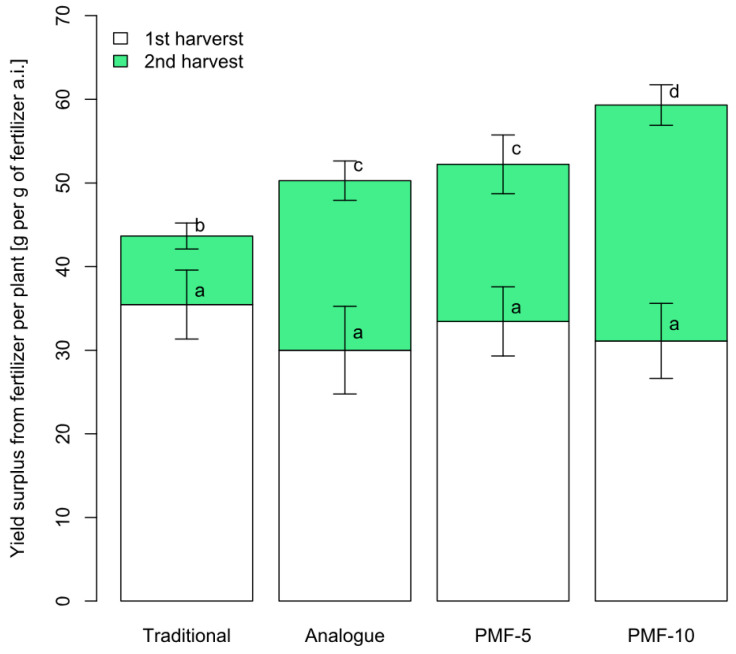
Average yield surplus from fertilizers in different nutrition systems. Different letters indicate statistically significant differences between the mean yield values at a confidence level of 0.95 (sample size for one system *n* = 9, with error bars showing the standard error of the mean).

The first harvest did not differ significantly between the nutrition systems. However, the yield of the second harvest showed notable differences. Figure 6 presents the yield increase (the difference in grams per plant from the control) for each nutrition system compared to the control. The effect of using all PMF systems was significantly higher compared to the traditional system, while application of PMF-10 was statistically greater compared to the commercial analogue and PMF-5. On average, the yield increase from PMFs was 2.8 times higher than the increase from the traditional fertilization system. In the analogue system, the yield increase was 2.5 times compared to the traditional variant. For the PMF-5 system, the increase was 2.3 times, while for the PMF-10 system, it was 3.4 times.

#### 3.1.3. Characteristics of Fertilizers

**Figure 7:**
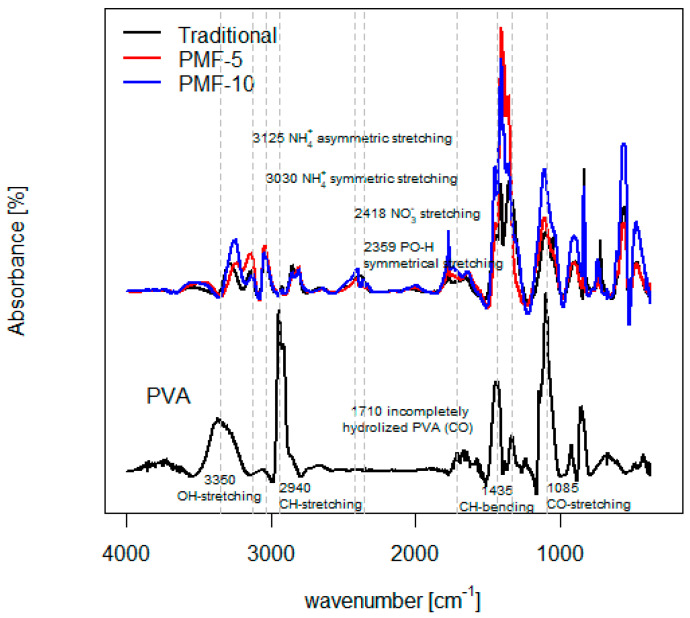
FT-IR absorbance spectra of PVA and CRF samples.

Fourier transform infrared (FT-IR) spectroscopy was used to verify the functional groups of polyvinyl alcohol (PVA), mineral fertilizers, and their blends. As can be seen in Figure 7, PVA showed a characteristic broad band between 3000 and 3600 cm^−1^, which refers to strong interaction of –OH intramolecular and intermolecular –H bonding with the maximum at about 3350 cm^−1^ (O–H stretching). Other bands indicative of PVA are 2940 cm^−1^ (C–H stretching) and 1435 cm^−1^ (C–H bending). PVA is primarily produced from polyvinyl acetate through the hydrolysis (saponification) of acetate groups. The degree of hydrolysis of PVA affects the properties of the resulting PVA; higher degrees of hydrolysis lead to increased crystallinity and thermal stability of PVA but lower water solubility. Bands typical of incompletely hydrolyzed PVA are seen at 1710 cm^−1^ and around 1085 cm^−1^ arising from stretching of the C–O bond [56,57].

Among the fundamental vibrational modes of the NH_4+_ group, there is a ν_1_ symmetric stretch at approximately 3030 cm^−1^ and a ν_3_ asymmetric stretch at approximately 3125 cm^−1^. Bands around 2418 cm^−1^ are associated with nitrate N-O stretching vibrations, and bands around 2359 cm^−1^ correspond to orthophosphate PO-H symmetrical stretching in mineral fertilizers [58,59,60]. These features are clearly visible in the FT-IR spectra of original mineral fertilizers and PMFs, distinctly contrasting with the spectra of PVA, which do not exhibit these bands.

Overall, the structures of PMFs were strongly confirmed by FT-IR spectroscopy by comparing the spectra of PVA, mineral fertilizers, and their blends, which have been attributed to their characteristic functional groups. The FT-IR results indicated that PMF-5 and PMF-10 indeed differ in composition according to their PVA content. The peaks characteristic of PVA, such as those around 1435 cm^−1^ and 1085 cm^−1^, were more pronounced in the spectrum of fertilizer with higher PVA content.

**Figure 8:**
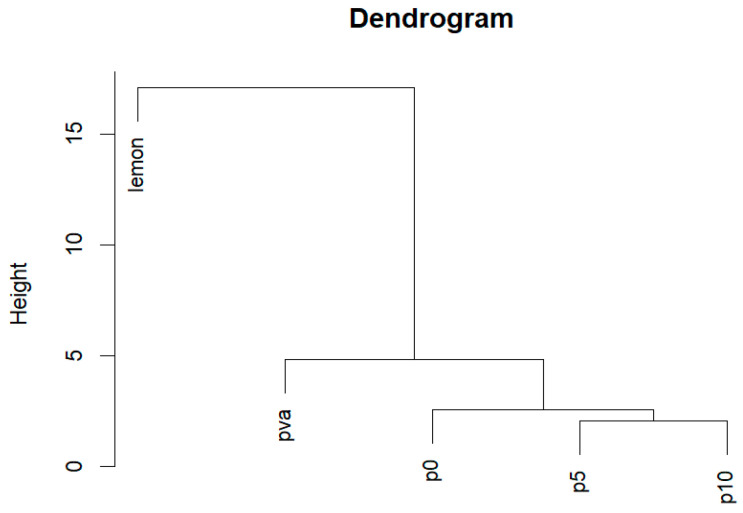
Hierarchical clustering dendrogram using Minkowski distance as metrics of dissimilarity between spectra of lemon acid, PVA, unmodified Azofoska (p0), and polymer-modified Azofoska with 5% (p5) and 10% (p10) of PVA.

Additionally, the dendrogram of all spectra, generated using Minkowski distance, demonstrates the degree of similarity and difference in the chemical composition of the samples (Figure 8). The spectra of PVA and the fertilizers showed similarities among themselves, with the spectra of all fertilizers being closer to each other than to the spectrum of pure PVA. At the same time, the spectra of the polymer-modified fertilizers were more similar to each other than to the spectrum of the original unmodified fertilizer.

#### 3.1.4. PMF Dissolution Rates

**Figure 9:**
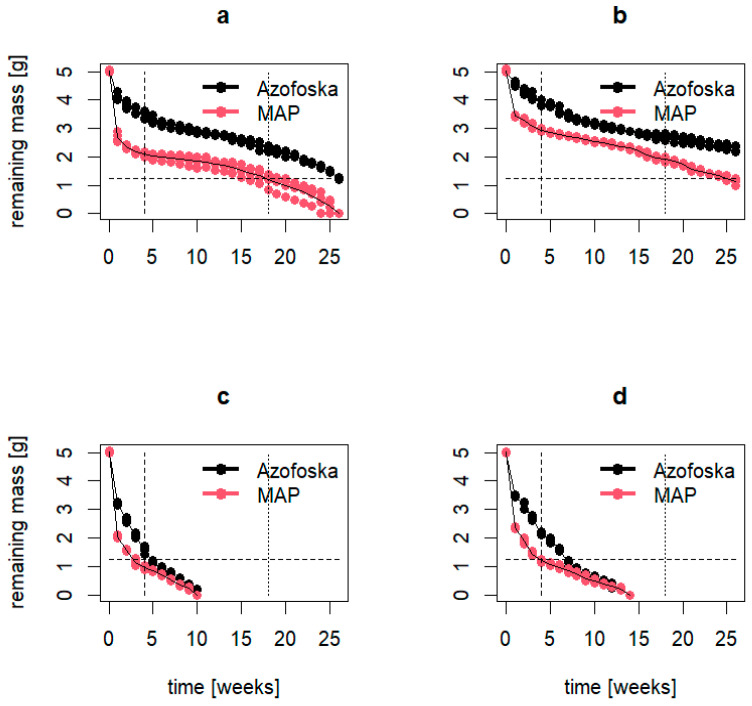
Dissolution rates of polymer-modified Azofoska and MAF, the components of PMF-5 and PMF-10 nutrition systems: (**a**) PMFs with 5% polymer content in laboratory; (**b**) PMFs with 10% polymer content in laboratory; (**c**) PMF with 5% polymer content in greenhouse and (**d**) PMF with 10% polymer content in greenhouse conditions.

Figure 9 illustrates the dissolution dynamics of PMF Azofoska and MAP tablets used in the PMF-5 and PMF-10 nutrition systems. The results indicate that, under laboratory conditions at a constant temperature of 25 °C, complete dissolution of the PMF tablets was not achieved even after 25 weeks. In contrast, under greenhouse conditions with natural temperature fluctuations averaging around 35 °C, complete dissolution occurred in 10 weeks for PMF-5 and in 14 weeks for PMF-10. Consequently, by the end of the strawberry field experiment (127 days or 18 weeks), it was assumed that all nutrients from the fertilizers had been released into the soil, and PMFs did not have any advantages further in the experiment in terms of dissolution rates.

#### 3.1.5. Soil EC

**Figure 10:**
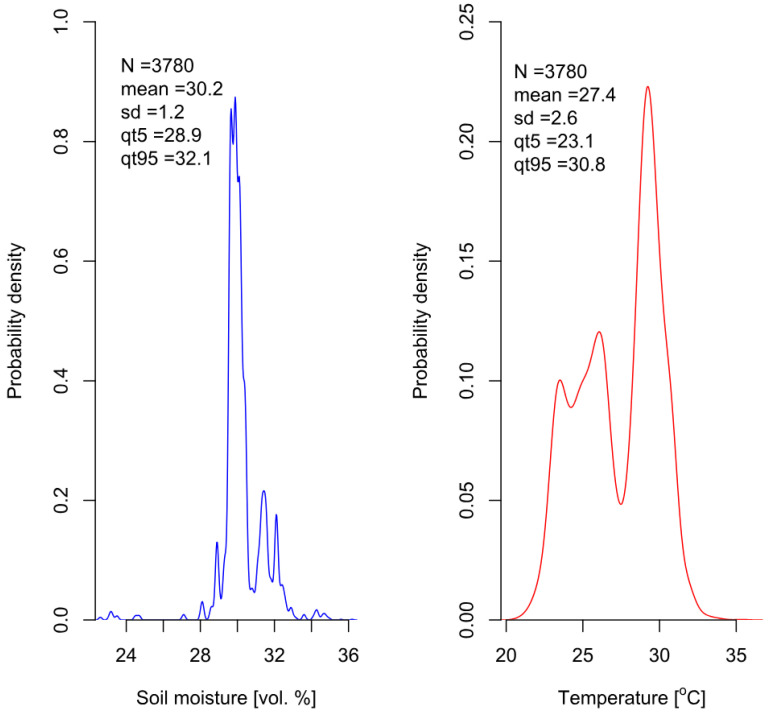
Probability densities for soil moisture (**left**) and temperature (**right**) during the measurement of soil EC throughout the duration of the experiment.

Soil EC depends on its moisture content and temperature. Therefore, all three parameters were measured simultaneously during the experiment. Soil EC values were corrected using temperature and moisture experimental data. The results indicate that soil volumetric moisture remained within a narrow range, fluctuating within 2% around the average value of 30%. It suggests uniform irrigation, thereby excluding moisture as a factor contributing to differences between the experimental variants. The probability density plot of soil moisture, based on 3780 measurements, is presented in Figure 10. Nevertheless, moisture data were used to standardize soil electrical conductivity values and convert them to the electrical conductivity of the soil solution, accounting for fluctuations in moisture. In contrast, the distribution of soil temperature values was multimodal, ranging between 20 and 35 degrees Celsius because the air temperature in the greenhouse was not fully controlled and it significantly increased during the growing season.

Soil EC values were corrected for temperature to a standard temperature of 25 °C according to the equation:

(1) $$EC_{25}=EC_{T}\times f_{T}$$

where *f_T_* is the temperature correction factor, calculated using the most effective model by Corwin and Lesch (2005) [61,62]:

(2) $$f_{T}=0.4470+1.4034e^{-T/26.815}$$

To correct for soil moisture, the soil pore water or soil solution EC (*EC*_w_) was calculated using the model proposed by G. Amente, John M. Baker, and Clive F. Reece [63]:

(3) $$EC_{w}=EC_{b}\theta^{-p}$$

where *EC_b_* is the temperature-corrected soil electrical conductivity, *θ* is the volumetric soil moisture expressed as a fraction, and p is the exponent that defines the tortuosity coefficient of the pore space (*θ^p^*). Parameter *p* is calculated similarly to gas diffusion models in soils and was set equal to 0.583 according to this model.

*EC_w_* is a measure of the ability of water in soil pores to conduct electric current. The conductivity of pore water is an important parameter in soil science, as it reflects soil salinity and nutrient availability for plants. *EC_w_* is the best indicator of the salinity status of the soil for plants, as it shows the level of salts that the plant roots actually encounter [54].

**Figure 11:**
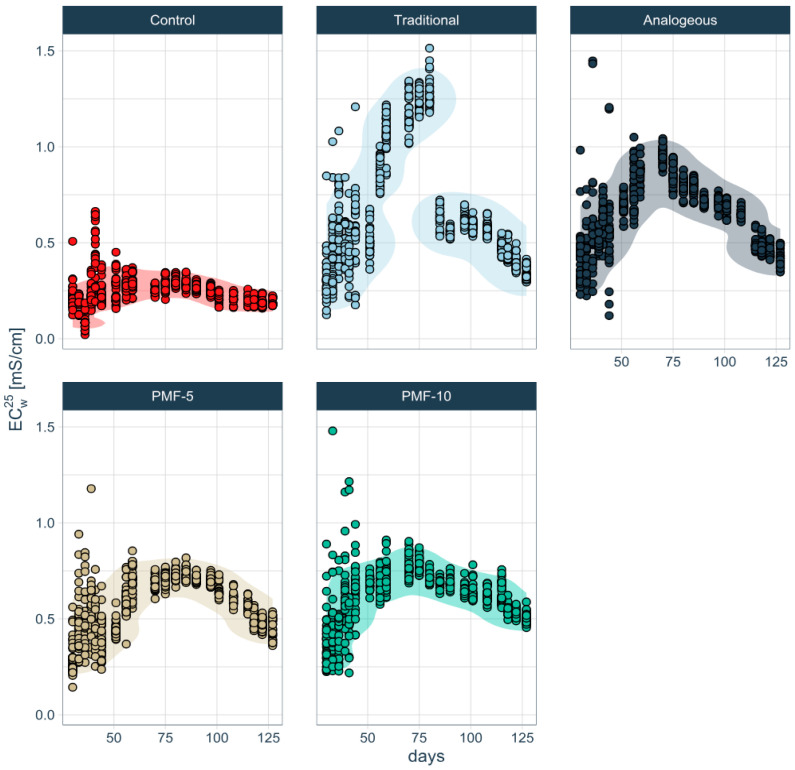
Time series of soil pore water EC under different fertilization systems, obtained by the correction for temperature and moisture. The corridor shows maximum density and encompasses 90% of the experimental data.

Analysis shows that all experimental variants exhibited EC profiles with a peak, significantly exceeding control values (Figure 11). This indicates that fertilizers dissolve during the growing season. However, the time pattern of fertilizer dissolution and changes in EC differ markedly between the traditional system and the three PMF systems.

In the traditional fertilization system, a sharp increase in soil electrolyte concentration was observed during the first instance of strawberry filling and ripening, peaking at approximately 1.5 mS/cm. This was followed by a significant decline of more than twofold, dropping to around 0.6 mS/cm right before the first harvest. In contrast, the differences among the three PMF systems are less pronounced. Among them, the analogue PMF had the highest soil solution EC values, peaking at up to 1 mS/cm, while the developed PMF-5 and PMF-10 showed EC values below 0.8 mS/cm. Additionally, during the formation of the second harvest, a gradual decrease in soil EC was noted in the analogue system, unlike in PMF-5 and PMF-10.

#### 3.1.6. Analysis of Differences in Soil EC Series PCA

**Figure 12:**
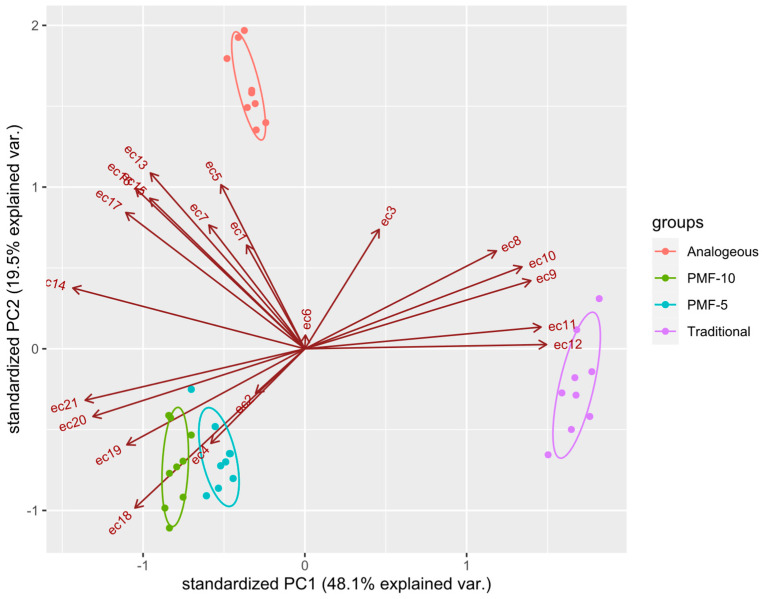
Clustering of conductivity data using PCA with 95% confidence intervals depicted with ellipses. ecN are the values of EC measured at time point N.

To assess the significance of differences in EC data, a PCA was performed. The results presented in Figure 12 indicate that the analogue system significantly differed from the PMF-5 and PMF-10 nutrition systems. Control data were excluded from the analysis due to their obvious differences from the others, while the traditional system was used as a reference point.

The PCA revealed that all PMF systems differed significantly from the traditional system, which was also evident from the overall shape of their EC dynamics, as seen in Figure 11. However, the PCA further showed that the PMF systems differed significantly from each other, a distinction that was not apparent from the graphical analysis alone.

The PCA results suggest that two factors, discussed above, primarily separate the PMF systems in the principal component space:

- the amplitude of the overall maximum EC at measurement points ranges from 7 to 10 (ec7–ec10), reflecting the period of the highest rates of dissolution;
- the stepwise decrease in EC during the later measurement points ranges from 17 to 21 (ec17–ec21), corresponding to the formation of the second harvest.

Both PMF-5 and PMF-10 nutrition systems showed significant differences but were located close to each other in the PCA plot, which was expected since they only differ in the polymer content.

#### 3.1.7. Assessment of Salt Quantity and Losses

**Figure 13:**
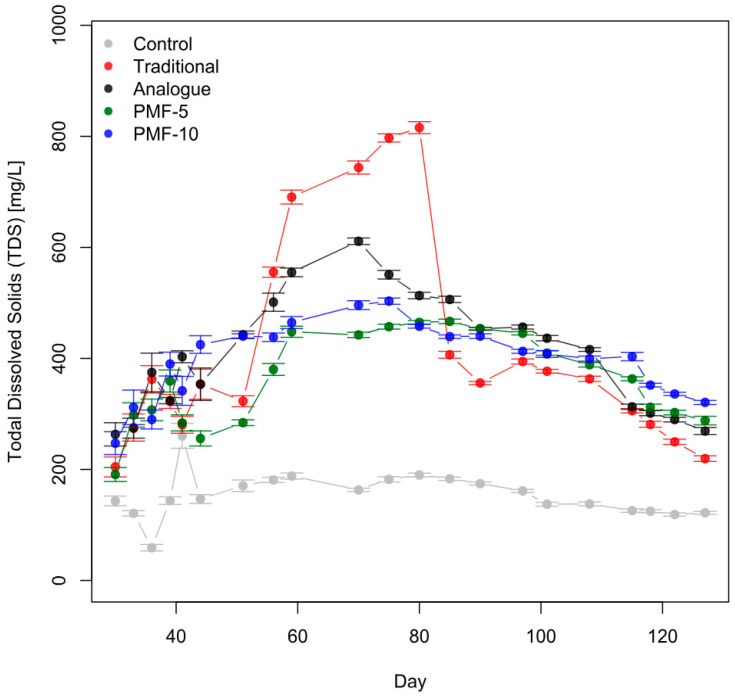
Dynamics of the total dissolved solids in the soil solution across all experimental variants, estimated based on the results of measurements of soil EC, volumetric moisture, and temperature. The average values are shown with the standard error of the mean (with 36 observations at each point, including all varieties as replicates).

Soil solution EC can be converted to the total dissolved solids (TDS) content in mg/L using a linear coefficient of 640 within the ECw range of 0.1–5 mS/cm [64]. Figure 13 shows the mean TDS values for all experimental variants. During the second berry filling period or after the second blossom (from approximately day 115 onwards), the salt content in the soil solution for the PMF-10 variant systematically exceeded the values in the other experimental variants, with the traditional system having the lowest TDS levels.

By the final measurement point (11 August 2023), the average TDS in the soil solution differed statistically significantly across all experimental variants, as confirmed by *t*-tests. The final measured TDS values in the PMF-10 variant exceeded those of the traditional fertilization system by 65%, PMF-5 by 41%, and the analogue system by 23%.

## 4. Discussion

### 4.1. Non-Productive Losses of Nutrients and Plant Use Efficiency

The initial soil analysis revealed high levels of plant-available phosphorus and average levels of plant-available potassium, which is considered insufficient for optimal strawberry cultivation. This created conditions that favored the positive effects of mineral fertilizers, particularly potassium. Although the traditional nutrition system showed significantly higher nutrient levels in the soil solution during the first yield formation period, it did not result in the highest yield. The lack of significant differences in first yields across the various nutrition systems indicates that the maximum strawberry yield was achieved under the given growing conditions.

The pronounced decline in salt content in the traditional nutrition system, a drop of approximately 500 mg/L in the soil solution, suggests non-productive salt losses. With a soil moisture content of around 30%, this equates to approximately 0.17 g of salts per kg of soil, accounting for about 10% of the applied fertilizers only in this one episode. The leaching of mineral salts from the soil is a well-recognized problem that impacts the effectiveness of mineral fertilizer use [65]. Since the first harvest did not show statistically significant differences among the experimental variants (with the exception of the control), the nutrient uptake was approximately the same. However, by the onset of the second harvest formation period, the soil EC level in the traditional variant had decreased to the lowest value, indicating more substantial non-productive losses compared to the other experimental variants. These observations support the rationale for utilizing prolonged-release fertilizers to reduce fertilizer losses.

Moreover, by the end of the experiment, the highest concentration of nutrients in the soil was observed in the following order of PMF-10 > PMF-5 > Analogue > Traditional (Figure 13). This indicated a slower release of nutrients from PMFs and, considering the higher second harvest yields in PMF systems, their more efficient utilization by the plants. This confirms our working hypothesis.

Although higher yields are typically associated with increased nutrient uptake, the final nutrient concentrations in the soil were also higher in the PMF variants. However, this may be not only the result of smaller non-productive losses in PMFs, but also to some extent, which is difficult to separate, due to the shifts in soil fertility status. It is known that the application of prolonged-release fertilizers enhances the mobilization of soil resources, such as phosphorus and potassium, transforming them into those forms that are more readily accessible to plants. This, in some cases, may lead to a positive long-term effect, reflected in an increased soil fertility levels of these nutrients [9,44,46].

### 4.2. Root Burn Stress

The efficiency of fertilizer utilization by plants and the underlying reasons for yield differences can be analyzed based on the second harvest, which exhibited significant variation among the experimental variants. During this second growth period, the levels of nutrients available to the plants also differed, as evidenced by the dynamics of soil EC. Although the visual differences in the conductivity profiles were relatively small, statistically significant differences were identified, favoring the PMF systems. Therefore, the higher second yields observed in the PMF systems can be attributed to the higher levels of nutrients throughout the second growth period.

It is important to note that under normal conditions, the second harvest of remontant strawberry varieties significantly exceeds the first. Therefore, the observed increase in the second strawberry harvests is important, despite its relatively small absolute magnitude in this experiment. In this study, the amount of applied fertilizers was adjusted to a level calculated for a single harvest. This enabled us to achieve variations in nutrient availability during the second yield formation and examine the differences in those yields avoiding the plateau effect, where yields no longer increase with additional mineral fertilizer applications, as was observed in the first harvest.

**Figure 14:**
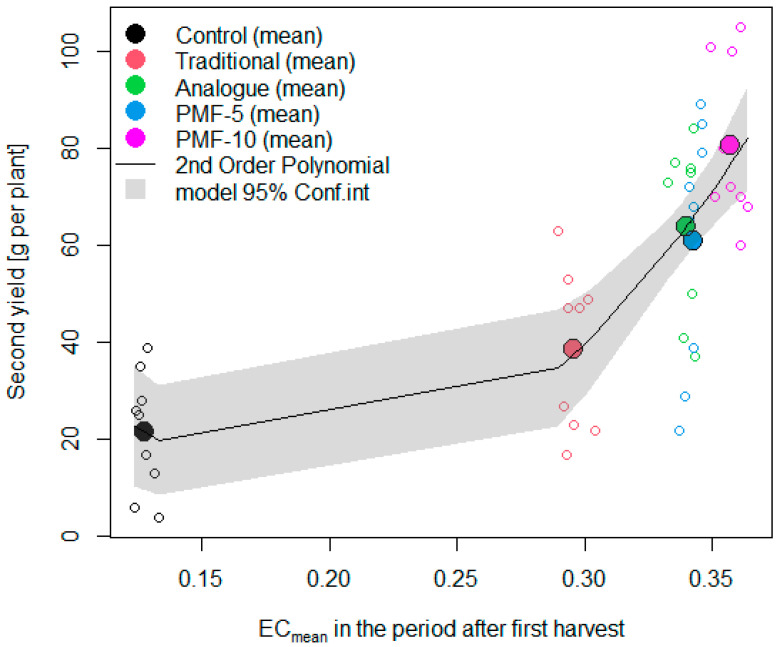
The relationship between the increase in the second harvest from fertilizers and the level of soil electrical conductivity relative to the control during the formation of the second harvest.

Generally, the level of nutrients has a linear effect on yield before it reaches a plateau. We analyzed the relationship between the second harvest and the average soil EC values during the period from the second flowering to the end of the measurements (Figure 14). This indicator serves as an integral characteristic, reflecting the total amount of nutrients available to the plants for the formation of the second harvest, which should have explained the yield differences observed among the experimental variants.

Despite the expected linear relationship between yield and nutrient levels within the studied range, the results indicated that the actual relationship was nonlinear. The coefficients of the linear model were far from being statistically significant, while the coefficients of the second-order polynomial were significant at a high level of significance (*p* < 0.001). Deviations from the linear model indicate the presence of an additional stress factor for the plants in the traditional, analogue, and PMF-5 fertilization systems, relative to the highest-yielding variant PMF-10. Figure 14 suggests a particularly pronounced plant stress for the traditional nutrition system, where the yield relative to the linear model was reduced nearly twofold.

**Figure 15:**
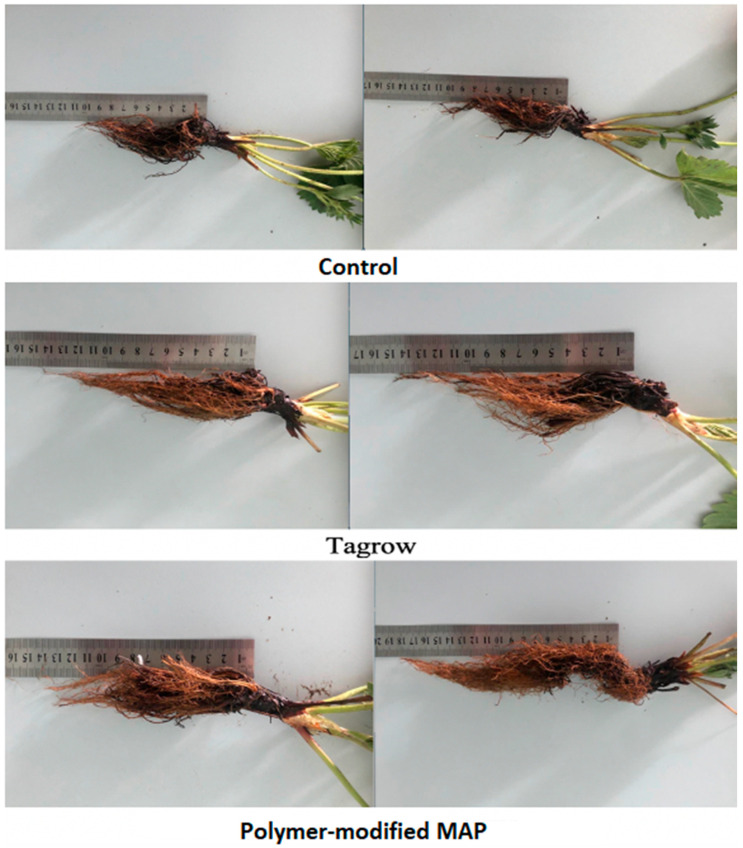
Root lengths of the strawberry variety Elizaveta with different nutrition (each shown in two replicates): control (10–11 cm), TAGROW CRF (13–14 cm), MAP PMF with polymer content 10% (15–18 cm).

This corresponds with the spike in EC observed during the formation of the first harvest in a traditional system. Such an EC spike may cause so-called “root burn” stress, as strawberries are a very sensitive crop. The literature describes the effect of reduced root biomass in strawberries with increasing soil EC [22]. There are also opinions that safe soil EC values during the seedling phase should be less than 0.35 mS/cm, and after flowering, no more than 0.35 mS/cm [66]. Unfortunately, there is insufficient research in the literature focused on studying this effect in the cultivation of various crops, highlighting the need for specialized experiments. In our case, the soil EC values in the traditional nutrition system during the growing season exceeded the levels discussed in the literature as safe. In our preliminary study in 2021, it was also found that with the traditional system the strawberry root development was indeed weaker based on its size [52], as shown in Figure 15.

Thus, to achieve high second harvests of remontant strawberries, a single application of PMF at the beginning of the season with sufficient nutrients for the second harvest can be a safe way of one-time mineral fertilizer application for the roots. Due to low non-productive losses, this will require approximately one and a half times the usual dose. In contrast, in the traditional nutrition system fertilizers need to be applied separately for each harvest to avoid uncontrolled losses and ensure the necessary nutrient levels for the second harvest. However, as we show, this is less efficient and increases operational costs. Moreover, as our study suggests, even a single dose of traditional forms of mineral fertilizers can lead to plant root stress from high spikes of rapidly soluble fertilizers, negatively affecting the second harvest.

## 5. Conclusions

The hypothesis regarding the effectiveness of mineral fertilizers through the slow release of nutrients from PMFs explains the observed differences in the second yield, where surplus from fertilizers was on average 2.8 times higher than in the traditional fertilization system. These studies, using 3780 sets of parallel measured time series for soil EC, moisture, and temperature, showed that the use of PMFs helps to reduce non-productive losses of mineral fertilizers. As a result, in the case of remontant garden strawberries, the amount of mineral nutrients in the soil for the subsequent harvest remains at a higher level (mean soil ECw values were about 20% higher for PMFs in the second period), ensuring a greater yield without the need for additional fertilizer application. In the end of the experiment, final measured TDS values in the soils with PMF variants exceeded those of the traditional fertilization system by up to 65%.

Furthermore, it was found that non-productive losses during the dissolution of traditional complex NPK fertilizer can result in elevated salt concentrations in the soil solution, with spikes in the range of 1 to 1.5 mS/cm. For highly sensitive crops such as garden strawberries, this creates a risk of plant stress in the form of “root burn.” This stress, in turn, leads to poor root system development, adversely affecting the second harvest, which shows disproportionately low yield relative to nutrient levels, i.e., the yield compared to the linear model was reduced by nearly half.

Thus, the application of PMFs represents an effective and optimal strategy for cultivating remontant strawberries. Moreover, even a slight increase in the second harvest of garden strawberries when using PMFs can significantly enhance the overall productivity of the crop and fertilizer efficiency under conditions of limited mineral fertilizer application.

## Data Availability

All materials and datasets related to this publication are accessible to the readers.
